# Use of dedicated gene panel sequencing using next generation sequencing to improve the personalized care of lung cancer

**DOI:** 10.18632/oncotarget.8391

**Published:** 2016-03-26

**Authors:** Coureche Guillaume Kaderbhai, Romain Boidot, Françoise Beltjens, Sandy Chevrier, Laurent Arnould, Laure Favier, Aurélie Lagrange, Bruno Coudert, François Ghiringhelli

**Affiliations:** ^1^ Department of Medical Oncology, Centre Georges-François Leclerc, Dijon, France; ^2^ INSERM, U866, Faculté de Médecine, Université de Bourgogne and Centre Georges François Leclerc, Dijon, France; ^3^ Department of Biology and Pathology of Tumors, Centre Georges-François Leclerc, Dijon, France; ^4^ Platform of Transfer in Cancer Biology, Centre Georges-François Leclerc, Dijon, France

**Keywords:** precision medicine, lung cancer, NGS, clinical research

## Abstract

Advances in Next Generation Sequencing (NGS) technologies have improved the ability to detect potentially targetable mutations. However, the integration of NGS into clinical management in an individualized manner remains challenging. In this single-center observational study, we performed a dedicated NGS panel studying 41 cancer-related genes in 50 consecutive patients with metastatic non-small-cell lung cancer between May 2012 and October 2014. Molecular analysis could be performed in 48 patients with a good quality check. One hundred and thirty-three mutations, whose twenty-four unique mutations, were detected. At least one mutation was found in 46 patients. In 58% of cases, the Molecular Tumor Board (MTB) was able to recommend treatment with a targeted agent based on the evaluation of the tumor genetic profile and treatment history. Nine patients (18%) were subsequently treated with a MTB-recommended targeted therapy; four patients experienced a clinical benefit with a partial response or stabilization lasting more than 4 months. In this case series involving patients with metastatic non-small cell lung cancer, we show that including integrative clinical sequencing data into routine clinical management was feasible and could impact on patient therapeutic proposal.

## INTRODUCTION

Lung cancer is the leading cause of cancer-related death worldwide [1]. First-line therapy comprises platinum-based chemotherapy and is subsequently followed by second-line cytotoxic chemotherapy. This strategy leads to median progression-free survival of approximately 1 year [2, 3]. Activating mutations of the *EGFR* gene are found in a subset of lung carcinomas (10% of adenocarcinomas in the Caucasian population) and define a subpopulation of cancers that can benefit from oral EGFR tyrosine kinase inhibitors (TKIs) [4]. Randomized phase III clinical trials have demonstrated that targeting *EGFR* mutations with these EGFR TKIs as the first-line treatment improves progression-free survival (PFS) and overall survival compared with chemotherapy [5-9]. Accumulating evidence demonstrates that in addition to *EGFR* mutations, other mutations such as *echinoderm microtubule-associated protein-like 4* (*EML4*) gene fusion to the *anaplastic lymphoma kinase* (*ALK*) gene, or *c-Met* gene amplification or *ROS1* gene rearranged or *ERBB2* exon 20 mutations could be targeted by dedicated targeted therapy with meaningful clinical efficacy [10-12]. While the above shows that knowledge of tumor genetic profiles is now extremely important to inform treatment decisions, the increasing number of targetable genes raises the problem of detecting mutations using a simple and fast dedicated genetic test.

NGS (Next Generation Sequencing) analysis of tumor cell DNA was developed for this purpose. It has provided physicians with a genomic map of cancer cells and could ease the access to targeted therapy, especially in NSCLC (non-small-cell lung cancer). In this report, we present the experience of our center, where 50 patients with NSCLC underwent NGS analysis. The results were discussed by the Molecular Tumor Board (MTB) to interpret genetic alterations and guide treatment.

## PATIENTS AND METHODS

### Tumor preparation and DNA extraction

Fifty formalin-fixed paraffin-embedded tumors from patients treated at the Centre Georges-François Leclerc between May 2012 and October 2014 were characterized by a pathologist to determine the tumor cell content and sent to the molecular biology platform for DNA extraction. Pathological slides were reviewed with the local pathologist for all patients. Blue alcian staining and immunohistochemistry were used to test the expression of p63 and TTF1 for each patient. All samples harbored a tumor cell content superior to 30%, avoiding microdissection experiments. Seven 15μm tumor slices were extracted using the Maxwell 16 FFPE Plus LEV DNA purification kit (Promega, Madison, USA) according to the manufacturer's instructions. DNA quality was assessed by spectrophotometry with absorbance at 230, 260, and 280 nm. DNA was quantified using a fluorimetric assay with a Qubit device.

The DNA quantity range was from 500 ng to 1.5 μg, and the DNA quality (260/280) was superior to 1.6 for 48 analyzed samples. For 2 samples, DNA quantity was inferior to 150 ng, and the 260/280 ratio was inferior to 1.2.

### Library preparation and sequencing

Libraries were prepared with the Truseq Custom Amplicon kit (Illumina, San Diego, USA) and sequenced as described previously [13]. For the design, the DNA target size was around 250 bp. Briefly, 500 ng of gDNA in 5 μl water were hybridized with an oligo pool. Then, unbound oligos were removed, and extension-ligation of bound oligos was followed by PCR amplification. PCR products were cleaned and checked for quality using Tapestation analysis (Agilent). The PCR product size had to be around 350bp. Before sequencing, the libraries were normalized thanks to the normalization process of the Truseq Custom Amplicon kit.

Twelve samples were multiplexed for each run thanks to their specific index combination. Libraries were paired-end sequenced with 2*151bp cycles on a MiSeq device (Illumina).

### Bioinformatics, annotations and interpretation of the results

The obtained sequences were aligned to the human reference genome hg19 (BWA) and variants were annotated by GATK and Variant Studio software (Illumina). A genetic variant was defined by a Q-score above 30 (except for indel mutations). Every variant was checked manually by a molecular biologist with visualization on Golden Helix Genome Browser. Variants with a frequency above 10% with a coverage depth superior to 300X were retained. The mean coverage was not informative due to the amplicon technology, in opposition to capture technologies. The multiplexing of samples was performed to obtain a minimum of 300X of reads per nucleotide studied.

For each variant, public databases and the literature were searched to classify the effect, the function, and potential therapeutic impact. As described in Supplementary Table 1, variations were classified as loss of function, decreased activity, gain of function, SNP, or unknown. For the therapeutic impact, variants were classified as targetable when they were associated with FDA-approved drugs, potentially targetable when their location could be associated with a clinical trial or a potential sensitivity to a drug and not targetable when the location and impact were unknown. When 2 targetable mutations were present in a same sample, we recommended treating the alteration with the higher mutation signal, reflecting the majority clone in the tumor.

### Validation of observed mutations

Mutations observed in NGS, occurring in genes analyzed in routine diagnosis (for solid tumor) were confirmed by allelic discrimination (*KRAS* mutations on codons 12 and 13, *EGFR* mutations on codons 790 and 858), fragment analysis (*EGFR* deletions for exon 19), and Sanger sequencing (*EGFR* mutations not routinely tested, *BRAF* mutations, *KIT* mutations, *PIK3CA* mutations, *ALK* mutations, and *TP53* mutations). We listed the mutations detected by NGS strategy and confirmed them with standard technics (Supplemental Table 1).

### Routine testing for lung cancer

Routine testing was performed in an independent platform for the analysis of *BRAF codon 600*, *KRAS* codons 12 and 13, and *PIK3CA* codons 542 and 545 *by allelic discrimination. EGFR* exons 18 (G719A/C/S), 20 (T790M) and 21 (L858R and L861Q) mutations were analyzed by allelic discrimination, *EGFR* exons 19 and 20 insertion/deletion analysis was performed by fragment analysis. In case of low input DNA, these exons were analyzed by Sanger sequencing. We listed the mutations detected by standard technics in the routine lab (Supplemental Table 1).

### Organization of the molecular tumor board: from suggestion to conclusion

**Figure 1:**
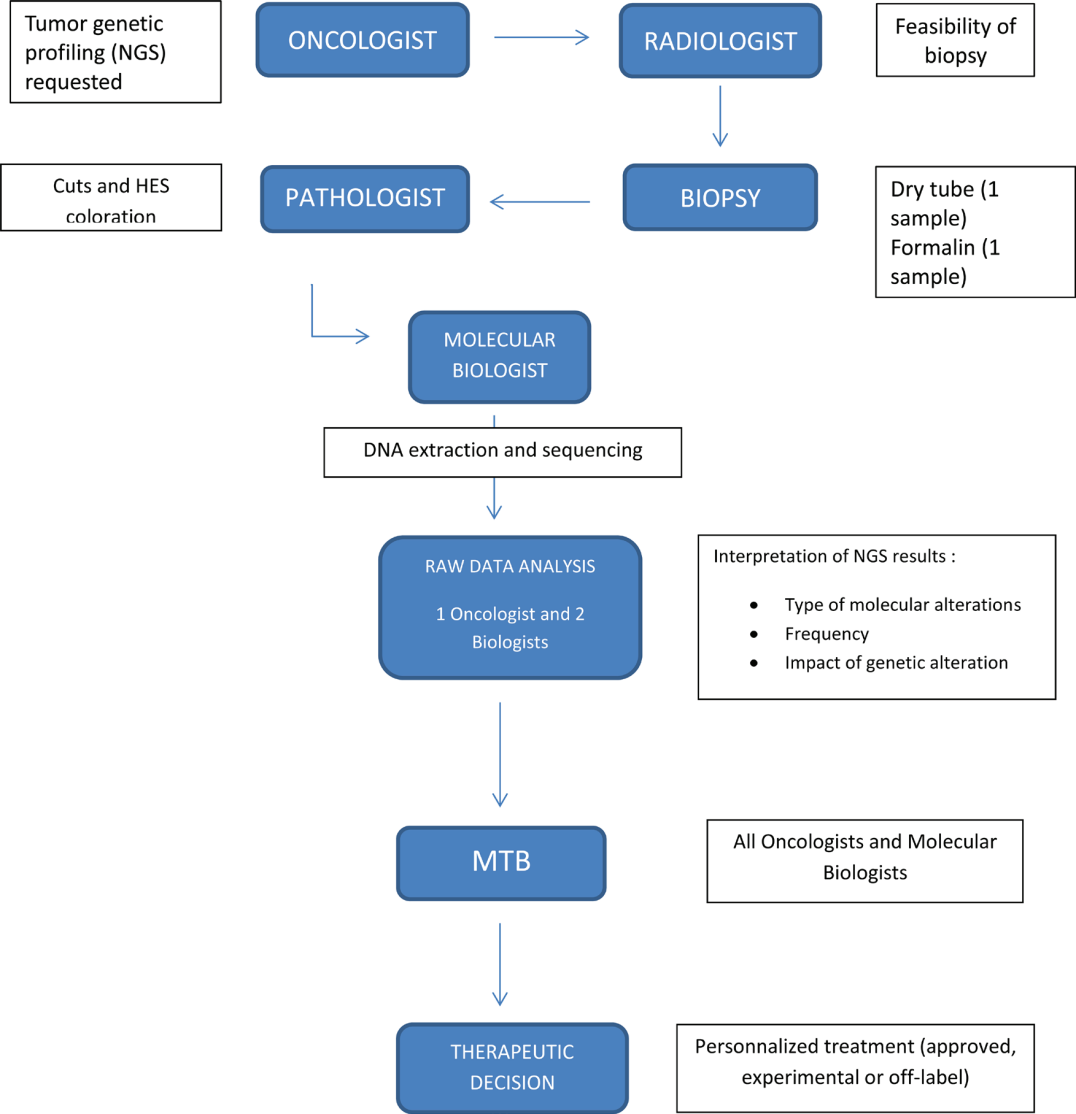
MTB, from suggestion to conclusion Abbreviations: NGS, Next Generation Sequencing; MTB, Molecular Tumor Board; HES, Hematoxilin Eosine Saffron.

The decision to evaluate a tumor's genetic profile was initially requested by the patient's consultant oncologist after oral consent. Analysis was done on the paraffin embedded tumor sample used for the diagnosis or on a new dedicated sample if there was no tissue available. The annotation of the detected variants for each gene indicated the exon, nucleotide, impact at the protein level, and frequency of the variation. The impact of the protein variation on protein function was determined by using data obtained from bibliography and public databases. We classified variations into five different classes: unknown, single-nucleotide polymorphism (SNP), decreased activity, loss-of-function, and activating mutation (Supplementary Table 1). Data analyses were then reviewed by an oncologist and two molecular biologists in order to provide a clinical interpretation of the variations detected. The therapeutic proposal was based on data from the literature, from clinical trial articles, case reports and *in vitro* or *in vivo* research (murine models). In cases where the impact of the mutation was unknown, the therapeutic proposal was based on the location of the mutation in the protein and on bioinformatics predictions of structural changes in protein conformation. After this therapeutic proposals were presented to the Molecular Tumor Board (MTB) (Figure 1). These proposals could be: i) inclusion in an early clinical trial, ii) use of a targeted therapy in their classical approval or iii) use of an approved drug in a new indication dictated by the molecular variation.

## RESULTS

### Patients' characteristics

NGS analysis of tumor cell DNA was performed on 50 consecutive patients with unresectable locally advanced or metastatic NSCLC. The population was balanced for gender since there were 26 women (52%) and 24 men (48%). The most common histological type was adenocarcinoma (82%, *n* = 41), followed by squamous-cell carcinoma (6.0%, *n* = 3), large-cell neuroendocrine carcinoma (4.0%, *n* = 2), undifferentiated carcinoma (4.0%, *n* = 2), papillary adenocarcinoma (2%, *n* = 1) and sarcomatoid carcinoma (2%, *n* = 1). Twenty-nine (58%) patients were smokers or former smokers, 19 (38%) had never smoked and 2 (4%) had an unknown smoker status. There were 6 patients with locally advanced NSCLC and 44 with metastatic tumors. The sample for NGS analysis was obtained using core needle biopsy of the lung tumor for locally advanced tumor. For other patients the sample was obtained from either primary tumor (21 cases) or metastases (liver in 15 cases, lymph nodes in 6 cases and adrenal tumors in 2 cases). The median age at NSCLC diagnosis was 62.7 years. The patients' clinical characteristics are presented in Table 1. Before NGS analysis, routine molecular testing recommended by the French National Cancer Institute was performed. All patients were tested for *EGFR*, *KRAS*, *BRAF*, *PIK3CA* and *ERBB2* by allelic discrimination, fragment analysis or Sanger sequencing. *ALK* rearrangement, *cMET* amplification and *ROS1* rearrangement were analyzed by immunohistochemistry and FISH. Among the 50 patients, 24 (*n* = 48%) harbored a variant revealed by routine molecular testing. The most common variant was an *EGFR* mutation found for 13 patients (9 patients with a deletion in exon 19, and 4 patients with an L858R mutation in exon 21. Two patients harbored a concomitant T790M mutation in exon 20). Five other patients had a *KRAS* mutation, two patients had a *BRAF* mutation, and four had *cMET* amplification (without a mutation). When possible, mutation detected by NGS analysis were confirmed by routine technic.

**Table 1:** patients' characteristics

Characteristic	Treatment-naive patients	Pretreated patients	Total
Sex, No. (%)			
Female	5 (41.7)	21 (52.6)	26 (52.0)
Male	7 (58.3)	17 (47.4)	24 (48.0)
Age at diagnosis, years			
Median	60.5	63.3	62,7
Range	42-78	20-79	20-79
ECOG performance status, No. (%)			
0	4 (33.3)	4 (10.5)	8 (16.0)
1	3 (25.0)	19 (50.0)	22 (44.0)
2	5 (41.7)	13 (34.2)	18 (36.0)
≥ 3	0 (0.0)	2 (5.3)	2 (4.0)
Cigarette smoking history, No. (%)			
Never smoked	5 (41.7)	14 (36.8)	19 (38.0)
Former or current smoker	7 (58.3)	22 (57.9)	29 (58.0)
Unknown	0 (0.0)	2 (5.3)	2 (4.0)
Histology, No. (%)			
Adenocarcinoma	10 (83.3)	31 (81.6)	41 (82.0)
Squamous cell carcinoma	0 (0.0)	3 (7.9)	3 (6.0)
Other	2 (16.7)	4 (10.5)	6 (12.0)
Specific mutation before NGS No. (%)			
EGFR	5 (41.7)	8 (21.1)	13 (26.0)
KRAS	1 (8.3)	4 (10.5)	5 (10.0)
BRAF	1 (8.3)	1 (2.6)	2 (4.0)
Other	0 (0.0)	4 (10.5)	4 (8.0)
No mutation	5 (41.7)	21 (55.3)	26 (52.0)
Number of lines of treatment			
Median	1.5	2.6	2.3
Range	1-3	1-7	1-7

### NGS analysis revealed new molecular variations

**Figure 2:**
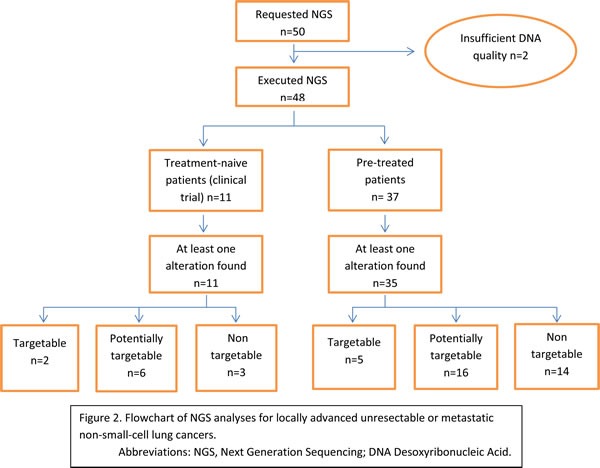
Flowchart of NGS analyses for locally advanced unresectable or metastatic non-small-cell lung cancers Abbreviations: NGS, Next Generation Sequencing; DNA Desoxyribonucleic Acid.

NGS analyses were requested by a consultant oncologist either at diagnosis of the NSCLC, in treatment-naive patients (22%, *n* = 11) as part of an observational study (ALCAPONE study NCT02281214), or after at least one line of treatment (chemotherapy or targeted therapy) (78%, *n* = 39) in order to find a new therapeutic option due to treatment failure and disease progression. Only two analyses could not be performed due to poor DNA quality probably because of the size of the tumor samples (bronchial aspiration) which results in a small amount of cells inducing a low DNA quantity and higher contaminant content. Figure 2 represents the flow chart and the detail of the NGS results (Figure 2).

**Figure 3:**
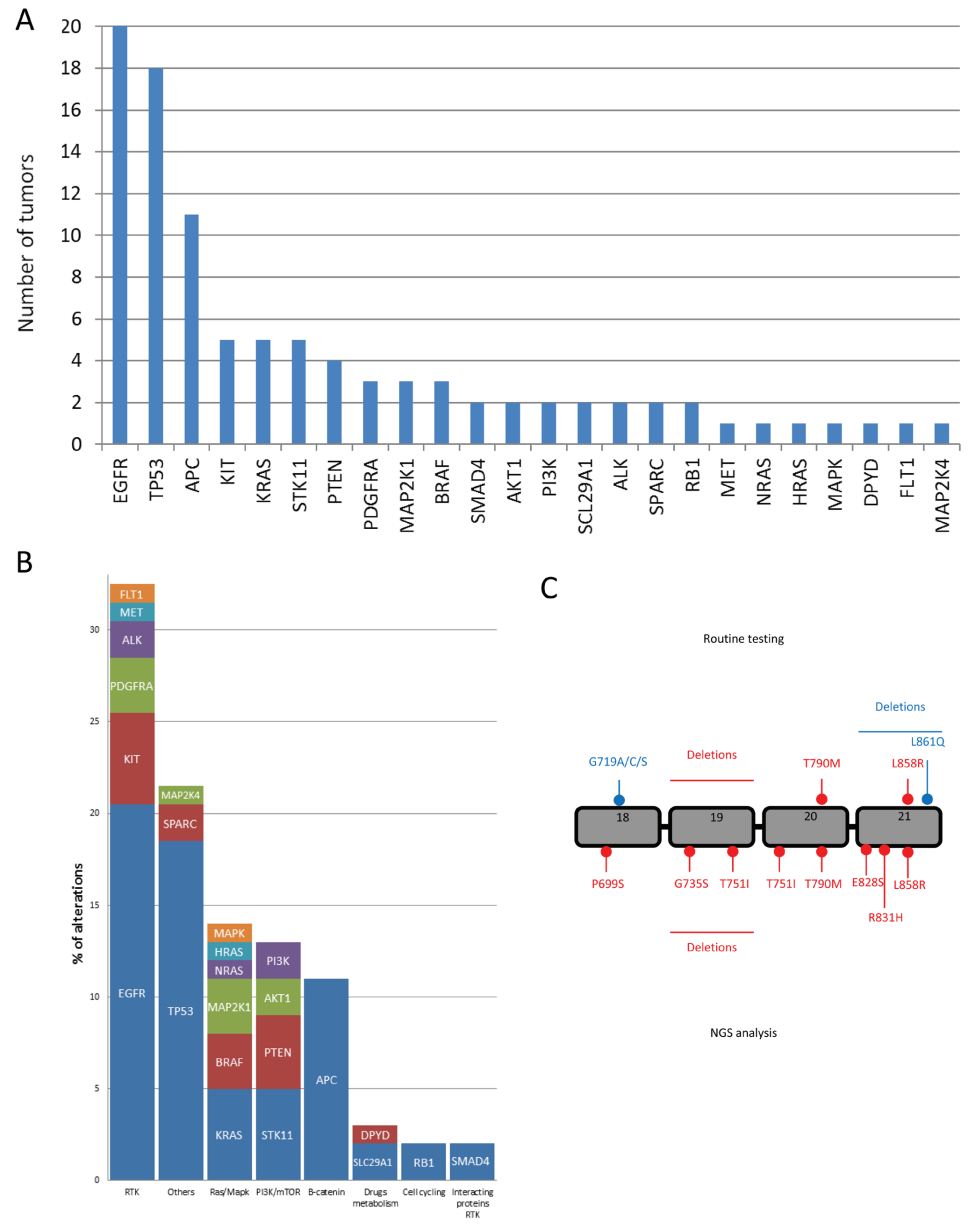
Mutations discovered using NGS panel **A.** Number of tumors with mutation, **B.** Distribution of mutations per signaling pathways. **C.** Representation of *EGFR* gene with the localization of EGFR mutation detected with routine testing and EGFR mutation detected with NGS panel.

Among the 48 tumors analyzed in this cohort, we detected 124 different mutations. There was a median of two molecular variations per patient (range: 1-14 variations). We detected at least one variation in 46 patients. Interestingly, no patients harbored the same variation profile. The genes with the highest mutation rate were *TP53* (26 mutations observed in 26 different patients), *APC* (18 mutations observed in 15 patients); *EGFR* (23 mutations observed in 20 patients) (Figure 3A). These mutations could be grouped in main signaling pathways underlining that gene encoding Tyrosine Kinase Domain Receptors were the most frequently mutated genes (Figure 3B). We detected six mutations in the *EGFR* gene in unusual locations, not searched in routine testing (Figure 3C). Five patients were reported to have somatic STK11 mutations. No clinical phenotype of Peutz-jegher's syndrome was detected in these patients and no germline mutation were detected.

Among the 124 molecular variations detected, bibliographic analysis found 34 targetable mutations in 29 patients. These therapeutic proposals were presented at the MTB. Table 2 summarizes the targetable variants with MTB recommendation and outcome.

**Table 2:** Treatments recommended by the MTB and implemented or not in patients

Patients	Mutation	Specific Mutation	Treatment recommended in MTB	Followed treatment /Line of therapy	PFS (Months)
1	AKT activating mutation / KIT activating mutation	L28F / T594I	mTOR inhibitor or Imatinib	Standard treatment (chemotherapy Platin-Pemetrexed)/1	
2	ALK	R1279K	Crizotinib	**MTB treatment (Crizotinib)/1**	**3**
3	KRAS	G12V	Experimental trial with Selumetinib	Standard treatment (chemotherapy Platin-Pemetrexed-Bevacizumab) /1	
4	BRAF	G466E	Experimental trial (Acsé Vemurafenib)	**MTB treatment (Acsé Vemurafenib)/1**	**3**
5	EGFR activating mutation	UncommonG735S	TKI EGFR	**MTB treatment(Gefitinib)/2**	**7Partial response**
6	EGFR activating mutation	UncommonL828S	TKI EGFR	**MTB treatment(Afatinib)/2**	**3**
7	EGFR activating mutation	UncommonR831H	TKI EGFR	**MTB treatment(Afatinib)/2**	**5Partial response**
8	EGFR activating mutation	ClassicalE746_A750del	TKI EGFR	**MTB treatment(Erlotinib)/1**	**5Partial response**
9	EGFR activating mutation / RB1 loss of function	ClassicalA747_T751del /L694X	TKI EGFR	BSC/3	
10	EGFR activating mutation / STK11 loss of function	ClassicalE746_A750del /Leu201AlafsX64	TKI EGFR or mTOR inhibitor	Standard treatment (chemotherapy Platin-Pemetrexed)/1	
11	EGFR	UncommonP699S	TKI EGFR	BSC/3	
12	MAP2K1	P232L	MEK inhibitor	**MTB treatment(Trametinib)/3**	**unevaluable**
13	STK11 loss of function	E256X	mTOR inhibitor	Standard treatment (chemotherapy Docetaxel)/2	
14	STK11 loss of function	L201AfsX6	mTOR inhibitor	Standard treatment (chemotherapy Pemetrexed)/2	
15	MAP2K1	P232L	MEK inhibitor	Standard treatment (chemotherapy Platin-Pemetrexed)/1	
16	KIT activating mutation	H630Y	Imatinib	**MTB treatment(Imatinib)/3**	**unevaluable**
17	KRAS activating mutation	G12C	Experimental trial with Selumetinib	Standard treatment (chemotherapy Gemcitabine)/3	
18	KRAS activating mutation	G13C	Experimental trial with Selumetinib	BSC/3	
19	KRAS activating mutation	G12A	Experimental trial with Selumetinib)	BSC/2	
20	PDGFRA activating mutation	R554S	Imatinib	**MTB treatment/2**	**5Partial response**
21	PDGFRA activating mutation	M642I	Imatinib	BSC/3	
22	PDGFRA activating mutation / PTEN loss of function	Y555C /R159K	Imatinib or Experimental trial with PI3K inhibitor	BSC/3	
23	PI3K activating mutation	IVS9+1	Experimental trial with PI3K inhibitor	BSC/3	
24	PI3K activating mutation	H994Y	Experimental trial with PI3K inhibitor	BSC/3	
25	PTEN loss of function	K62TfsX34	Experimental trial with PI3K inhibitor	BSC/3	
26	PTEN loss of function	S229X	Experimental trial with PI3K inhibitor	BSC/2	
27	PTEN loss of function	E201K	Experimental trial with PI3K inhibitor	BSC/2	
28	STK11 loss of function	G279AfsX8	mTOR inhibitor	Experimental trial with anti PDL1/2	
29	STK11 loss of function / KRAS activating mutation	R333C/G12C	mTOR inhibitor or clinical trial with Selumetinib	Standard treatment (chemotherapy Platin-Pemetrexed)/1	

Abbreviations : BSC, Best supporting care; TKI, Tyrosine kinase inhibitor

### Patients' follow-up and outcomes

The median time between the request for a molecular diagnosis and the presentation of a therapeutic proposal by the MTB was 20 calendar days (range: 10 - 62 calendar days). A dedicate biopsy was required for 21 patients. The mean delay from biopsy to MTB decision was 25 days (range: 15-41 calendar days). Half of patients were studied by the MTB while they were still under therapy so that an alternative plan could be prepared for implementation at the time of progression. Of the 24 patients who were still responding to their previous treatment, 18 showed subsequent disease progression. A proposal was given for 11 patients and three of these initiated the treatment proposed by the MTB.

For the 24 other patients presented, the previous treatment had already failed and a proposal was provided by the MTB in 18 cases and initiated in six patients.

To date, treatment decisions according to the molecular results have been followed in nine patients. For the other patients (*n* = 20), the treatment was not based on the MTB proposal because patients were stable on their previous treatment (*n* = 1), or another classical treatment decision was preferred (because of the cost of molecular targeted therapy, or the patient was not eligible for the clinical trial) (*n* = 7); or because of a quick deterioration in the patient's performance status or death (*n* = 12).

Nine patients received treatment according to MTB, 3 in first line, 4 after failure of first line and 2 after failure of second line. Four showed a partial response for at least 4 months. Mean progression-free survival was 4.5 months. Two out of the three patients treated with anti EGFR therapy for rare mutation discovered by NGS (and not detected by classical testing) and one patient with classical EGFR mutation, experienced partial response. A patient treated with imatinib for *PDGFRa* mutation also responded to this targeted therapy.

## DISCUSSION

In the case of NSCLC, a number of driver alterations like mutations, gene translocations or amplifications that can benefit from targeted therapies, have been discovered in the past ten years [14] [10-12, 15-17]. As a consequence, the tumor molecular status needs to be known before the first-line therapy because these mutations dictate the use of targeted therapies rather than classical chemotherapies. The accumulation of targetable mutations increases the complexity of the analyses carried out at the diagnosis of metastatic diseases, and delays the beginning of therapy. In addition, dedicated molecular testing currently recommended by the French National Cancer Institute does not capture all targetable mutations. Consequently, it appears logical to propose Next Generation Sequencing for lung cancer patients to search for other genomic alterations that could be targetable. We report our experience in using an NGS strategy that includes discussion of cases by a MTB. This strategy is a resource for clinicians as it helps them to interpret genetic profiles and to implement anticancer recommendations. Here, we used a dedicated panel of genes and could test 41 genes at once. In our study, NGS revealed 133 genomic variants in a total of 50 patients. All of the patients but two had at least one genomic mutation. One of the pitfalls of this strategy is that such NGS panel performed only on tumor cells could not make the difference between germline and somatic mutations. However most genes in this panel are targetable oncogenes for which mutations were essentially somatic.

This strategy has the capacity to detect non-canonical variants that may potentially be actionable, rather than routine molecular testing which only focuses on well-known actionable variants. This was particularly important for the *EGFR* gene, for which we found six actionable variants not detected using routine testing. Classical mutations of the *EGFR* gene include exon 19 deletions of 15-18 pb, which represent more than 50% of *EGFR* mutations, and the exon 21 point mutation at the residue L858R, which represents more than 30% [18]. In addition, routine analysis revealed L861Q and G719 mutations, which confer modest sensitivity to EGFR TKI [19-21]. In addition to these classical mutations, other rare mutations with various degrees of sensitivity to EGFR TKI have been described [22-25].

Several trial designs are now incorporating genomic information identified through NGS methods [26]. However, the integration of such technology in a practical, efficient, and value-added manner is not straightforward. Some reports are upcoming for American hospitals involving small and heterogenous population of patients with different cancer location [27-31]. While many clinical trials on this subject are in progress in European countries, no European hospital has reported their experience with such strategy in a daily clinical practice.

The organization of the MTB requires optimal organisation, mainly for the quick analysis and interpretation of data. In this study, the time between the genetic analysis and MTB meeting was less than 30 days for all patients. Despite recommendations for treatments based on molecular analysis, not all patients received the targeted therapy because it was difficult to enroll them in phase I clinical trials or because they were not eligible for clinical trials (e.g. brain metastasis are frequently an exclusion criterion in clinical trials) or because of the patients' or their physician's preference. The result of this was that only a small proportion of patients received the therapy recommended by the MTB. Among 50 patients, the MTB recommended therapy for 29 patients and only 9 received this therapy. In a similar report from the Dartmouth hospital in Lebanon, only 25% of patients received the treatment recommended by the MTB [29]. In the San Diego Moores Cancer Center, NGS analyses affected the cancer treatment in 35.3% of cases [30]. A team from Vanderbilt University also reported that 17.5% of patients (18 of 103) with tumor genetic profiling received targeted therapy [30]. In the case of lung cancer, Hagemann reported that only 11% of sequenced patients received therapy based on NGS testing [32]. These results are very similar to our results.

In conclusion, using an NGS panel to improve molecular testing is feasible in routine practice and the information obtained was clinically relevant and allowed the MTB to propose a therapeutic change in 18% of cases. Our experience in the use of an MTB is too short to determine the clinical benefit of such an approach, but the accumulated evidence suggests that this strategy will become routine in comprehensive cancer centers. A major issue is the low rate of patients that could be treated following the recommendations of the MTB because they were tested at an advanced stage and only received supportive care rather than targeted therapy. So we believe that such analysis should be performed at the diagnosis of the metastatic disease or just after the recurrence after the first line therapy if patients still have a good performance status. The strategy, however, needs to be standardized and algorithms for medical recommendations must be established. There is also a clear need to develop clinical trials to make sure that the use of target therapies based on genotyping by NGS really improves survival in cancer patients.

## SUPPLEMENTARY MATERIAL AND TABLE


