# Supplementary material for: Use of dedicated gene panel sequencing using next generation sequencing to improve the personalized care of lung cancer
Source: Oncotarget. 2016 Mar 26;7(17):24860–70. doi: 10.18632/oncotarget.8391 (PMC5029748; doi:10.18632/oncotarget.8391)
Supplement: Supplementary file 1 [file oncotarget-07-24860-s001.pdf]

Use of dedicated gene panel sequencing using next generation sequencing to improve the personalized care of lung cancer

Supplementary Material

Supplementary Table 1: List of mutations with biological annotations

Genes	Exons	Protein variation	Impact	Detected in routine analysis<br><br>In routine lab	Confirmed by classical technics<br><br>In the NGS facility
AKT1	3	IVS2-1<br><br>L28F	Unknown<br><br>Unknown	No<br><br>No	Not tested<br><br>Nos tested
ALK	23<br><br>25	P1213S<br><br>/<br><br>R1279K	Unknown<br><br>/<br><br>Unknown	No<br><br>/<br><br>No	Yes<br><br>/<br><br>Yes
APC	16	R727S<br><br>S874A<br><br>S900A<br><br>S948P (*2)<br><br>L1029F<br><br>P1052S<br><br>L1129S<br><br>E1242G<br><br>E1353A<br><br>D1498Y<br><br>D1512N<br><br>V1822D (H/O)<br><br>T1932I<br><br>P2170L	Unknown<br><br>Unknown<br><br>Unknown<br><br>Unknown<br><br>Unknown<br><br>Unknown<br><br>Loss of function (1)<br><br>Unknown<br><br>Unknown<br><br>Unknown<br><br>Unknown<br><br>SNP<br><br>Unknown<br><br>Unknown	No<br><br>No<br><br>No<br><br>No<br><br>No<br><br>No<br><br>No<br><br>No<br><br>No<br><br>No<br><br>No<br><br>No<br><br>No<br><br>No	Not tested<br><br>Not tested<br><br>Not tested<br><br>Not tested<br><br>Not tested<br><br>Not tested<br><br>Not tested<br><br>Not tested<br><br>Not tested<br><br>Not tested<br><br>Not tested<br><br>Not tested<br><br>Not tested<br><br>Not tested

		V2414I	Unknown	No	Not tested
		G2502S	Benign (2)	No	Not tested
		S2578L	Unknown	No	Not tested
		S2799A	Unknown	No	Not tested
ARAF	7	/	/	No	Not tested
	8	/	/	No	Not tested
	11	/	/	No	Not tested
	15	/	/	No	Not tested
BRAF	11	G466E	Loss of function (3)	No	Yes
	15	D594N	Loss of function (4)	No	Yes
		V600E	Activation (5)	Yes	Yes
CRAF	7	/	/	/	/
	11	/	/	/	/
	15	/	/	/	/
CTNNB1	3	/	/	/	/
DPYD	23	T981I	Unknown	No	Nos tested
ERCC1	7	/	/	/	/
	10	/	/	/	/
EGFR	18	P699S	Unknown	No	Yes
	19	G735S	Activation (6)	No	Yes
		E746_E749del	Activation (7)	Yes	Yes
		E746_A750del (*8)	Activation (8)	Yes	Yes
		A747_T751del	Activation (8)	Yes	Yes
		A747_P753del_Sins	Activation (8)	Yes	Yes
		T751I	Activation (9)	No	Yes
	20	M766L	Unknown	No	Yes

		T790M (*2)	Activation (10)	Yes	Yes
	21	E828S	Unknown	No	Yes
		R831H	Activation (7)	No	Yes
		L858R (*4)	Activation (8)	Yes	Yes
ERBB2	20	/	/	/	/
FGFR1	4	/	/	/	/
	5	/	/	/	/
FGFR2	7	/	/	/	/
	12	/	/	/	/
FGFR3	16	/	/	/	/
FGFR4	2	/	/	/	/
	5	/	/	/	/
	11	/	/	/	/
	14	/	/	/	/
	16	/	/	/	/
FLT1	19	A897V	Unknown	No	Not tested
	24	/	/	/	/
	30	/	/	/	/
HRAS	2	/	/	/	/
	3	Q99H	Unknown	No	Not tested
	4	/	/	/	/
IGF1R	2	/	/	/	/
	5	/	/	/	/
	6	/	/	/	/
	7	/	/	/	/
	8	/	/	/	/
	19	/	/	/	/

	20	/	/	/	/
JAK2	12	/	/	/	/
	14	/	/	/	/
	16	/	/	/	/
JAK3	5	/	/	/	/
	11	/	/	/	/
	12	/	/	/	/
	15	/	/	/	/
KDR	7	V297I (H)	Loss of function SNP (11)	No	Not tested
	11	Q472H (H/O)	Gain of function SNP (12)	No	Not tested
		C482R (H)	SNP	No	Not tested
		A505T	Unknown	No	Not tested
	16	G756D	Unknown	No	Not tested
		T771M	Unknown	No	Not tested
	24	P1074T	Unknown	No	Not tested
	30	A1295V	Unknown	No	Not tested
		P1354S	Unknown	No	Not tested
KIT	9	/	/	/	/
	10	M541L	SNP (13)	No	Yes
	11	Q575X	Loss of function	No	Yes
	12	T594I	Unknown	No	Yes
	13	H630Y	Unknown	No	Yes
		H650N	Unknown	No	Yes
	17	I789T	Unknown	No	Yes
		A814V	Unknown	No	Yes
KRAS	2	G12A	Activation (14)	Yes	Yes
		G12C (*2)	Activation (14)	Yes	Yes

		G12V	Activation (14)	Yes	Yes
		G13C	Activation (14)	Yes	Yes
	3	R68G	Unknown	No	Yes
	4	/	/	/	/
MAP2K1	2	P89L	Unknown	No	Not tested
	3	/	/	/	Not tested
	6	/	/	/	Not tested
	7	P232L	Unknown	No	Not tested
		P237R	Unknown	No	Not tested
	11	/	/	/	/
MAP2K2	1	/	/	/	/
	2	/	/	/	/
MAP2K4	3	IVS2-1	Unknown	No	Not tested
	4	/	/	/	/
	5	/	/	/	/
	8	R285S	Unknown	No	Not tested
		D289G	Unknown	No	Not tested
	9	/	/	/	/
MET	14	R988C	SNP (15)	No	Yes
		T1010I	SNP (15)	No	Yes
	16	/	/	/	/
	17	/	/	/	/
	18	/	/	/	/
	19	/	/	/	/
NRAS	2	/	/	/	/
	3	/	/	/	/
	4	P110L	Unknown	No	Not tested

PDGFRA	12	R554S	Unknown	No	Not tested
		R555C	Activation (16)	No	Not tested
	14	M642I	Unknown	No	Not tested
	18	/	/	/	/
PDK1	2	/	/	/	/
	5	/	/	/	/
	10	/	/	/	/
	11	/	/	/	/
PIK3CA	9	IVS9+1	/	No	Yes
	20	H994Y	Unknown	No	Yes
PTEN	3	K62TfsX34	Loss of function (stop codon)	No	Not tested
	5	R159K	Loss of function (17)	No	Not tested
	6	E201K	Unknown	No	Not tested
	7	S229X	Loss of function (stop codon)	No	Not tested
RB1	11	K359R	Unknown	No	Not tested
	20	L694X	Loss of function (stop codon)	No	Not tested
SLC28A1	7	V189I (H/O)	Loss of function SNP (18)	No	Not tested
SLC29A1	9	V291I	Unknown	No	Not tested
	10	/	/	/	/
SMAD4	10	/	/	/	/
	11	V437I	Unknown	No	Not tested
		A460T	Unknown	No	Not tested
SPARC	5	/	/	/	/
	6	/	/	/	/
	7	K191R	Unknown	No	Not tested
	9	T266S	Unknown	No	Not tested
		Y288H	Unknown	No	Not tested

SRC	14	/	/	/	/
STK11	4	/	/	/	/
	5	L201AfsX6	Loss of function (stop codon)	No	Not tested
		H202R	Unknown	No	Not tested
	6	E256X	Loss of function (stop codon)	No	Not tested
		G279AfsX8	Loss of function (stop codon)	No	Not tested
	8	R333C	Unknown	No	Not tested
TP53	4	IVS4+1	Unknown	No	Yes
		R72P	SNP (19)	No	Yes
		W91R	Loss of function (19)	No	Yes
	5	P142VfsX28	Loss of function (stop codon)	No	Yes
		G144X	Loss of function (stop codon)	No	Yes
		R158L	Loss of function (19)	No	Yes
		Y163C	Loss of function (19)	No	Yes
		C176F	Loss of function (19)	No	Yes
		S183X	Loss of function (stop codon)	No	Yes
	6	G187R	Loss of function (19)	No	Yes
		P191L	Loss of function (19)	No	Yes
		V216M	Loss of function (19)	No	Yes
		Y220C (*3)	Loss of function (19)	No	Yes
		Y220X	Loss of function (stop codon)	No	Yes
	7	I232N	Loss of function (19)	No	Yes
		Y234C	Loss of function (19)	No	Yes
		R248W	Loss of function (19)	No	Yes
		R249G	Loss of function (19)	No	Yes
		E258K (*2)	Loss of function (19)	No	Yes
	8	R273C	Loss of function (19)	No	Yes

		V274F	Loss of function (19)	No	Yes
		R282W	Loss of function (19)	No	Yes
	9	K319RfsX27	Loss of function (stop codon)	No	Yes
		D324N	Loss of function (19)	No	Yes
TYMS	4	/	/	/	/
	5	/	/	/	/
	6	/	/	/	/

1. Lefevre et al., Journal of Human Genetics – 2012
2. Schlussel et al., Case Reprots in Medicine – 2014
3. Wan et al., Cell – 2004
4. Heidorn et al., Cell – 2010
5. Davies et al., Nature – 2002
6. Cai et al., Oncogene – 2008
7. Yang et al., JCO - 2008
8. Lynch et al., NEJM – 2004
9. Tsao et al., NEJM – 2005
10. Kobayashi et al., NEJM – 2005
11. Tao et al., J Biol Chem - 2001
12. Glubb et al., Clin Cancer Res – 2011
13. Krüger et al., Leukemia – 2006
14. Scheffzek et al., Science – 1997
15. Tyner et al.,Cancer Res – 2010
16. de Raedt et al., Gastroenterology – 2006
17. Cecener et al., Cell Mol Neurobiol – 2009
18. Gray et al., Mol Pharmacol – 2004

19. Petitjean *et al.*, Human Mutation - 2007
